# Mesonephric-like Differentiation of Endometrial Endometrioid Carcinoma: Clinicopathological and Molecular Characteristics Distinct from Those of Uterine Mesonephric-like Adenocarcinoma

**DOI:** 10.3390/diagnostics11081450

**Published:** 2021-08-11

**Authors:** Sujin Park, Go Eun Bae, Jiyoung Kim, Hyun-Soo Kim

**Affiliations:** 1 Department of Pathology and Translational Genomics, Samsung Medical Center, Sungkyunkwan University School of Medicine, Seoul 06351, Korea; sujin423.park@samsung.com; 2 Department of Pathology, Chungnam National University School of Medicine, Daejeon 34134, Korea; goeunbae1@gmail.com; 3 Department of Pathology, Incheon St. Mary’s Hospital, College of Medicine, The Catholic University of Korea, Incheon 21431, Korea

**Keywords:** endometrium, endometrial cancer, endometrioid carcinoma, uterus, mesonephric-like adenocarcinoma, immunohistochemistry, targeted sequencing

## Abstract

When diagnosing endometrial carcinoma cases, we encountered histological features that strikingly resembled uterine mesonephric-like adenocarcinoma (MLA), but the differential diagnosis remained challenging after performing immunostaining. Considering the aggressive biological behavior and poor prognosis of uterine MLA, we believe that the accurate recognition of mesonephric-like differentiation (MLD) is important in the diagnosis of endometrial carcinoma. We aimed to investigate the clinicopathological and molecular characteristics of such cases and compared them with those of uterine MLAs. Five patients diagnosed with endometrioid carcinoma (EC) with MLD were included in this study. Histological evaluation, immunostaining, and targeted sequencing were performed. All five tumors showed typical morphological features of MLA, including densely aggregated tubular structures, deep basophilia under low-power magnification microscopy, eosinophilic intraluminal secretions, and diverse growth patterns. Immunostaining revealed moderate-to-strong nuclear immunoreactivity for estrogen and progesterone receptors in more than 50% tumor cells. The staining intensities and proportions of PAX2 and GATA3 were variable. None of the tumors harbored *KRAS* mutations. Considering the prognostic implications, ancillary tests, including immunostaining and targeted sequencing, should be performed to accurately differentiate between endometrial EC-MLD and uterine MLA.

## 1. Introduction

Endometrial carcinoma is the most common gynecological malignancy, and its incidence and mortality are increasing in developed countries [1]. Endometrioid carcinoma (EC) is its most common histological subtype, accounting for approximately 80% of all endometrial carcinomas [2,3]. The recognition of its typical morphology and clinical significance is usually straightforward. However, the various architectural and cytological features of EC can represent a diagnostic challenge and be associated with a malignancy of a more aggressive behavior [2].

Mesonephric adenocarcinoma (MA) is a rare malignancy of the female genital tract and is thought to be derived from the embryonal remnants of the mesonephric tubules and ducts [4,5,6,7,8,9,10,11,12,13,14] and accounts for less than 1% of all gynecological malignancies [15]. MA typically arises in the uterine cervix and vagina, but several cases of MA arising in the upper female genital tract have also been documented [4,6,7,8,10,15,16,17,18,19]. Since their association with mesonephric remnants has not been firmly established, MA of the uterine corpus and ovary has been referred to in the literature as mesonephric-like adenocarcinoma (MLA) [15,17]. It has recently been shown that uterine MLAs show more aggressive biological behavior and worse prognosis than other histological subtypes of endometrial carcinoma [4].

Recently, we encountered some cases of endometrial EC that exhibited histological and immunohistochemical features similar to those of uterine MLA. Although we considered the possibility of uterine MLA during the initial microscopic examination of these cases, information from their morphologies, immunophenotypes, and mutational profiles were insufficient for a definite diagnosis of uterine MLA. Instead, we diagnosed these cases as endometrial EC with mesonephric-like differentiation (EC-MLD). No diagnostic criteria for EC-MLD have been established to date, and the clinical significance of MLD in association with EC has not been documented in the literature. Furthermore, there have been no studies regarding the differences in clinicopathological and genetic features between endometrial EC-MLD and uterine MLA. Thus, in this study, we aimed to investigate the clinicopathological and molecular characteristics of five EC-MLD cases and compared them with those of uterine MLAs. Our observations of the clinical, histological, immunophenotypical, and genetic features of EC-MLD will help pathologists accurately recognize and diagnose this rare condition.

## 2. Materials and Methods

### 2.1. Case Selection

Using a combination of the keywords “endometrium”, “adenocarcinoma”, “endometrioid carcinoma”, and “mesonephric-like differentiation”, we found five cases from the surgical pathology archives of the Department of Pathology and Translational Genomics at the Samsung Medical Center (Seoul, Republic of Korea). The following clinical information was collected: age of the patient, menopausal status, previous history of gynecological conditions or breast cancer, presenting symptoms, magnetic resonance imaging (MRI) findings, preoperative serum levels of cancer antigen (CA) 125 and CA 19-9, histology of endometrial curettage, preoperative clinical impression, surgical procedure, initial pathological International Federation of Gynecology and Obstetrics (FIGO) stage [20], postoperative treatment, recurrence, and metastasis.

### 2.2. Pathological Examination

Two board-certified pathologists specializing in gynecological oncology reviewed all available hematoxylin and eosin (H&E)-stained slides via light microscopy. The following information was collected for pathological staging: histological subtype, greatest dimension and invasion depth of tumor, extension into the cervical stroma, uterine serosa, parametrium, vagina, and adnexa; focal (<5 vessels) or substantial (≥5 vessels) lymphovascular space invasion (LVSI); and metastasis to the peritoneum, lymph nodes, and distant organs. We also investigated the following histological and cytological features: intraluminal eosinophilic secretion; architectural diversity (tubular, ductal, papillary, solid, cystic, comedonecrosis-like, and sex cord-like), squamous and mucinous differentiation, endometrial atypical hyperplasia/endometrioid intraepithelial neoplasia (AH/EIN), nuclear pleomorphism, conspicuous nucleoli, mitotic count (per 10 high-power fields), atypical mitotic figure, and tumor cell necrosis.

### 2.3. Immunostaining

Four-micrometer-thick, formalin-fixed, paraffin-embedded sections were deparaffinized and rehydrated using xylene and alcohol solutions. Immunostaining was performed using automated instruments [7,16,21,22,23,24,25,26,27]. After antigen retrieval, the sections were incubated with primary antibodies against paired box 8 (PAX8, 1:50, polyclonal, Cell Marque, Rocklin, CA, USA), PAX2 (1:100, polyclonal, Invitrogen, Thermo Fisher Scientific, Waltham, MA, USA), GATA-binding protein 3 (GATA3, 1:400, clone L50-823, Cell Marque), Wilms tumor 1 (WT1, 1:800, clone 6F-H2, Cell Marque), estrogen receptor (ER, 1:150, clone 6F11, Novocastra, Leica Biosystems, Newcastle Upon Tyne, UK), progesterone receptor (PR, 1:100, clone 16, Novocastra), p16 (prediluted, clone E6H4, Ventana Medical Systems), p53 (1:300, clone DO-7, Novocastra), and phosphatase and tensin homolog deleted on chromosome 10 (PTEN, prediluted, clone SP218, Ventana Medical Systems). After chromogenic visualization, the sections were counterstained with hematoxylin. Appropriate positive and negative controls were stained concurrently to validate the staining method we used. Ovarian high-grade serous carcinoma (for PAX8, WT1, p16, and p53), endometrial endometrioid carcinoma (for ER, PR, and PTEN), and invasive breast carcinoma of no specific type (for GATA3) were used as positive controls. Negative controls were prepared by substituting non-immune serum with primary antibodies, resulting in no detectable staining. The proportion of staining was established as diffuse positive when at least 50% tumor cells were stained, focal positive when less than 50% tumor cells were stained, and negative if none of the tumor cells were stained. For PAX8, PAX2, GATA3, WT1, ER, and PR, staining in the nuclei was interpreted as a positive expression [7]. The intensity of staining was graded as strong, moderate, weak, or absent. For PTEN, weak staining in the cytoplasm was interpreted as preserved expression, whereas loss of expression was defined as the complete absence of cytoplasmic PTEN immunoreactivity. The p53 immunostaining pattern was interpreted as a mutation pattern when one of the following staining patterns was observed: diffuse and strong nuclear immunoreactivity in ≥75% tumor cells (over-expression pattern), no nuclear immunoreactivity in any of the tumor cells (complete absence pattern), or an unequivocal cytoplasmic staining (cytoplasmic pattern). In contrast, p53 expression was interpreted as a wild-type pattern if a variable proportion of tumor cell nuclei expressed the p53 protein with a mild-to-moderate staining intensity [28]. The p16 immunostaining pattern was interpreted as diffuse and strongly positive when its expression was continuous and strong either via nuclear or nuclear and cytoplasmic staining. All other p16 immunostaining patterns, described as focal nuclear staining, wispy, blob-like, puddled, or scattered cytoplasmic staining, were interpreted as patchy positive [7,16,29,30,31].

### 2.4. Targeted Sequencing and Data Analysis Pipelines

Total DNA and RNA were isolated from 10 μm-thick slices of FFPE tissue using a sterile 26-gauge needle and a RecoverAll Multi-Sample RNA/DNA Isolation Workflow (Thermo Fisher Scientific, Waltham, MA, USA). The tumor tissue was obtained through manual microdissection and subjected to DNA and RNA extraction for library preparation. Normal tissues from each case were obtained from the adjacent non-neoplastic area. DNA and RNA were quantified using a Qubit 2.0 Fluorometer (Thermo Fisher Scientific). DNA libraries were prepared as previously described [7,23,24,25,29,30,32,33]. These DNA libraries were generated from 20 ng of DNA per sample using an Ion AmpliSeq Library Kit 2.0 (Thermo Fisher Scientific) and an Oncomine Comprehensive Assay (OCA) v1 panel (Thermo Fisher Scientific). RNA libraries were generated from 15 ng of RNA per sample using the Ion AmpliSeq RNA Library Kit (Thermo Fisher Scientific). Libraries were quantified using the Ion Library Universal Quantification Kit (Thermo Fisher Scientific). The OCA v1 panel (Thermo Fisher Scientific) included 143 genes, 73 of which were interrogated for mutational hotspots and 26 tumor-suppressor genes were interrogated for all exons. The panel was able to detect copy number variations (CNVs) in 49 genes and fusion drivers in 22 genes. The complete gene list of the panel is available at https://www.thermofisher.com/kr/ko/home/clinical/preclinical-companion-diagnostic-development/oncomine-oncology/oncomine-cancer-research-panel-workflow.html, accessed on 31 July 2021. A 60 pmol/L pool of DNA:RNA libraries at a 4:1 ratio was used to prepare the templated ion sphere particles (Thermo Fisher Scientific). Sequencing was performed using the Ion 540™ Kit-Chef (Thermo Fisher Scientific) and Ion S5 system (Thermo Fisher Scientific). Sequencing data of approximately 200 bp reads were generated after 500 flow runs.

Sequencing data were analyzed using Torrent Suite Software v5.2.2 (Thermo Fisher Scientific). This workflow was created by adding a custom hotspot browser extensible data file to report mutations of interest and a custom CNV baseline (described below) using the manufacturer’s default workflow as described previously [32,33]. The pipeline included signal processing, base calling, quality score assignment, adapter trimming, read mapping to the human genome assembly GRCh37, quality control mapping, coverage analysis with down-sampling, and variant calling. The variants were identified using the Torrent Variant Caller plug-in and Ion Reporter Software v5.2 (Thermo Fisher Scientific). Coverage maps were generated using the coverage analysis plug-in (Thermo Fisher Scientific). Additionally, ANNOVAR (http://annovar.openbioinformatics.org/, accessed on 31 July 2021) was used for the functional annotation of identified single nucleotide polymorphisms (SNPs) to investigate their genomic locations and variations [34]. To eliminate error artifacts, sequence data were visually confirmed using the Integrative Genomics Viewer (Broad Institute, Cambridge, MA, USA). This workflow was able to report SNPs and indels in as low as 1% of the variant allele fraction. Based on the results of a feasibility study, the variant allele fraction threshold was set to 5%. Copy number analysis was performed using the copy number module within the aforementioned workflow of the Ion Reporter Software v5.2 (Thermo Fisher Scientific). Copy numbers of 4 or greater were considered concordant if the orthogonal assay also reported a copy number of 4 or greater for the target genes. Fusions were detected using the fusion detection module in the Ion Reporter Software (Thermo Fisher Scientific). This pipeline only reported fusions that were annotated previously, as defined in a reference file preloaded into the workflow [32,33].

## 3. Results

### 3.1. Case Presentation

This study included five patients diagnosed with EC-MLD. Here, we provide a brief description of the clinical presentation of each case as follows:

Case 1: A 54-year-old woman who had undergone hormone replacement therapy went to the outpatient clinic for follow-up visits. An endometrial mass was detected using ultrasonography. MRI also revealed a 2.9 cm linear soft tissue lesion in the endometrial cavity. The endometrial lesion appeared not to invade the deep myometrium. There was no evidence of peritoneal seeding, lymph node enlargement, or distant metastasis. The preoperative serum level of cancer antigen (CA) 125 was within the normal range (5.2). Under the clinical impression of endometrial cancer, she underwent total hysterectomy (TH) with bilateral salpingo-oophorectomy (BSO) and bilateral pelvic lymph node dissection (BPLND). She was diagnosed with stage IA grade 1 EC-MLD and did not receive any further treatment. The patient was alive without evidence of disease 15 months after the surgery.

Case 2: A 68-year-old woman presented with vaginal bleeding. A 3.3 cm endometrial mass was detected via MRI. Although some small separate endometrial lesions were noted adjacent to the mass, they appeared to involve only the superficial myometrium. No peritoneal seeding or metastasis was observed. Her serum CA 125 level was within the normal range (5.2 U/mL). Since her imaging findings suggested stage IA disease, TH with BSO and BPLND was performed. She was diagnosed with stage IA grade 1 EC-MLD and was followed up for 6 months without further treatment.

Case 3: A 57-year-old woman presented with vaginal bleeding. MRI revealed a 5.1 cm mass in the endometrial cavity, which invaded the inner half of the myometrium without peritoneal seeding or metastasis. Her serum CA 125 level was within the normal range (29.5 U/mL) before surgery. Under the clinical impression of stage IA endometrial cancer, she underwent TH with BSO and BPLND. Pathological examination revealed grade 2 EC measuring 5.5 cm in the greatest dimension and showed some foci of LVSI. Based on the presence of adverse risk factors, including large size and the presence of LVSI, she received adjuvant vaginal brachytherapy.

Case 4: The patient was a 67-year-old woman with multiple recurrent endometrial polyps. She had undergone surgery, chemotherapy, and radiation therapy (RT) for breast cancer 17 years previously and had been taking tamoxifen for five years. Regular follow-up ultrasonography revealed solid masses in both the endometrium and left adnexa. MRI revealed a 4.5 cm endometrial mass and a 4.4 cm lobulating adnexal mass. Her preoperative serum CA 125 level was elevated up to 134.7 U/mL. With the impression of endometrial cancer with ovarian or tubal extension, TH with BSO, BPLND, and para-aortic lymph node dissection (PALND) was performed. Pathological examination confirmed that the tumor involved the right fallopian tube (stage IIIA). The patient received postoperative whole pelvic RT. The patient was alive without evidence of disease 13 months postoperatively.

Case 5: A 56-year-old woman underwent MRI for abnormal vaginal bleeding. A huge uterine mass measuring 8.8 cm in the greatest dimension appeared to involve the cervical stroma and the full thickness of the myometrium. Serosal extension was suspected in the right posterior uterine wall, but there was no evidence of peritoneal seeding or metastasis. The radiologic impression was endometrial cancer with unusual histology or uterine sarcoma. Both her serum CA 125 (143.2 U/mL) and CA 19-9 (468.0 U/mL) levels were elevated. She underwent TH, BSO, PLND, and PALND for advanced endometrial cancer. Pathological examination confirmed serosal and ovarian extension, substantial LVSI, and para-aortic lymph node metastasis. The patient received concurrent chemoradiation therapy (CCRT) for stage IIIC2 disease.

### 3.2. Clinical Characteristics

**Figure 1:**
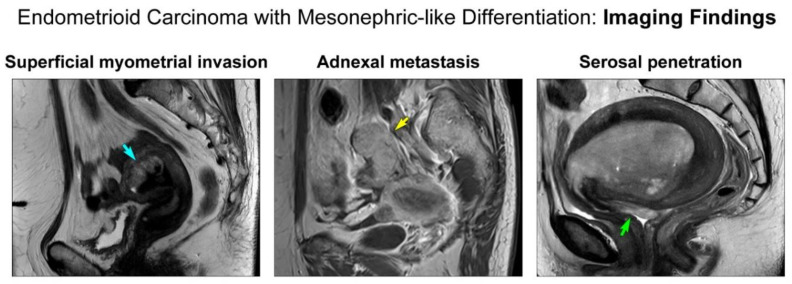
Preoperative magnetic resonance imaging findings. (**Left panel**) The tumor invades less than half of the myometrium (blue arrow). (**Middle panel**) The adnexa shows a metastatic tumor mass (yellow arrow). (**Right panel**) The tumor penetrates the uterine serosa (green arrow).

Table 1 summarizes the clinical features of these patients. All patients were postmenopausal women, and their ages ranged from 54 to 68 years (mean, 60.4 years). Two patients had a previous gynecological history. One patient underwent hormone replacement therapy (case 1). The other patient received surgery, chemotherapy, and tamoxifen for breast cancer and underwent more than one hysteroscopic polypectomy for recurrent tamoxifen-related endometrial polyps (case 4). These two patients were subjected to routine ultrasonographic follow-up, which revealed an endometrial mass (case 1) or both endometrial and adnexal masses (case 4). The other three patients presented with vaginal bleeding. Upon MRI, the mean size of the endometrial masses was 4.9 cm (range, 2.9–8.8 cm). In three patients (cases 1, 2, and 3), the endometrial masses invaded less than half of the myometrium (Figure 1) without adnexal, peritoneal, nodal, or distant metastases (MRI FIGO stage IA). In contrast, the remaining two patients were suspected to have MRI FIGO stage IIIA disease based on their adnexal (case 4; Figure 1) and serosal (case 5; Figure 1) extensions, respectively. The preoperative serum levels of CA 125 in these two patients were elevated to 134.7 U/mL (case 4) and 143.2 U/mL (case 5), respectively. The latter patient also had an increased serum CA 19-9 level (468.0 U/mL; case 5). Preoperative endometrial curettage specimens were interpreted as EC-MLD (2/5; cases 1 and 3), EC versus MLA (1/5; case 2), EC (1/5; case 4), and non-diagnostic (1/5; case 5). Based on the clinical impression of endometrial cancer, the patients underwent TH (5/5) with BSO (5/5), BPLND (5/5), and PALND (2/5). The initial pathological FIGO stages were IA (3/5; cases 1, 2, and 3), IIIA (1/5; case 4 with adnexal extension), and IIIC2 (1/5; case 5 with para-aortic lymph node metastasis). Among the three patients with stage IA disease, two (cases 1 and 2) did not receive any additional treatment, whereas one (case 3) underwent vaginal brachytherapy because she had the following risk factors for recurrence: large tumor size (5.5 cm) and LVSI. Two patients with advanced-stage EC received postoperative whole pelvic RT (case 4) and CCRT (case 5). Follow-up data were available for three patients (cases 1, 2, and 4). Two patients with stage IA disease (cases 1 and 2) and one with stage IIIA disease (case 4) were alive without evidence of disease at 18, 9, and 16 months postoperatively, respectively. Reliable follow-up information was unavailable for two patients (cases 3 and 5) because their postoperative follow-up period was less than three months. Both patients were still receiving postoperative treatment.

### 3.3. Pathological Characteristics

**Figure 2:**
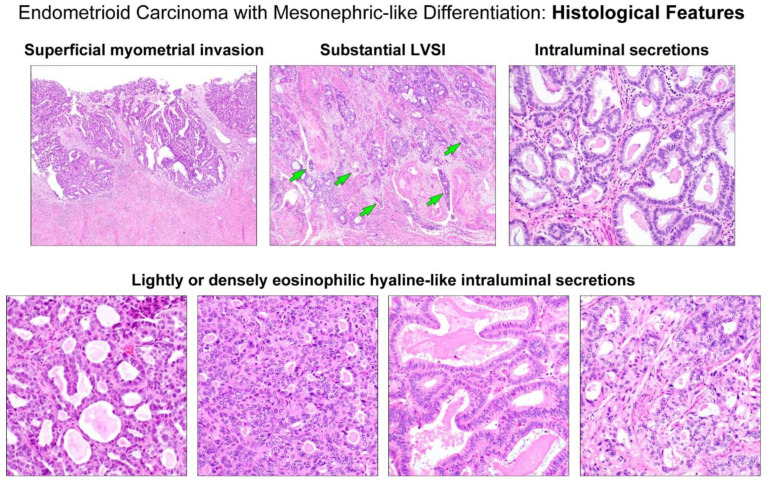
Histological features. (**Upper left panel**) The tumor invades less than half of the myometrium. (**Upper middle panel**) The invasive front displays substantial lymphovascular space invasion (LVSI; green arrows). (**Upper right panel**) The tubules and glands possess eosinophilic secretions. (**Lower panels**) Variable-shaped tubular and glandular lumina are filled with lightly or densely eosinophilic intraluminal hyaline-like secretions, characteristic of uterine mesonephric-like adenocarcinoma.

Table 2 summarizes the pathological features regarding the staging. The greatest dimension of the tumors ranged from 2.3–8.5 cm (mean, 4.8 cm). Four tumors (cases 1, 2, 3, and 4) invaded less than half of the myometrium (Figure 2, upper panel), with a mean invasion depth of 0.3 cm (range, 0.2–0.6 cm). One tumor (case 5) extended through the entire myometrial thickness into the uterine serosa. None of the tumors involved the cervical stroma, vagina, or parametrium. Two tumors showed LVSI, which were focal (case 3) and substantial (case 5; Figure 2, upper panel), respectively. Adnexal extension was identified in two cases (cases 4 and 5). The right adnexal mass detected via MRI was confirmed pathologically as a tubal extension of endometrial EC (case 4), and a single small focus (0.7 cm) of metastatic EC was identified in the left ovary (case 5). In case 5, a 0.5 cm metastatic tumor was also found in a single para-aortic lymph node. None of the patients had abdominal or pelvic peritoneal metastases. No distant metastases were reported until the last follow-up.

**Figure 3:**
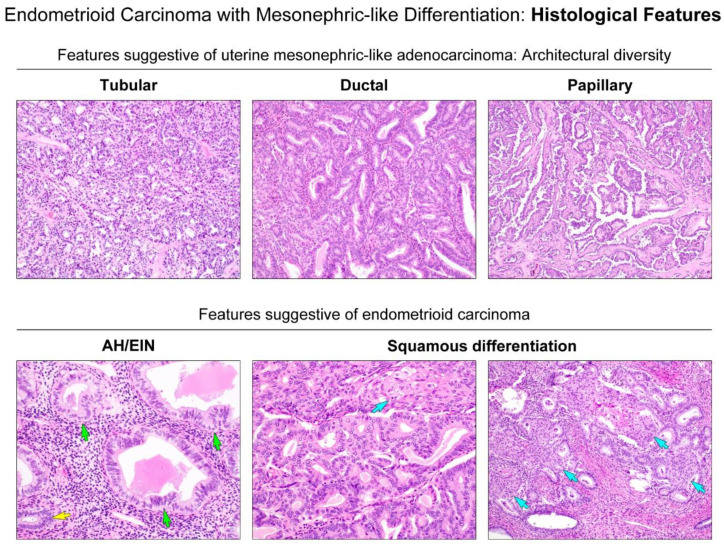
Histological features suggestive of either uterine MLA or endometrial EC. (**Upper panels**) Diverse growth patterns, including tubular, ductal, and papillary architecture, are suggestive of uterine MLA. (**Lower panel**) The presence of atypical hyperplasia/endometrioid intraepithelial neoplasia (AH/EIN) and foci of squamous differentiation (blue arrows) are suggestive of endometrial EC. Compared to the nuclei of non-atypical glands (yellow arrows), AH/EIN (green arrows) shows a definitive cytological demarcation, including nuclear pleomorphism, enlargement, rounding, and loss of polarity.

As summarized in Table 3, we further evaluated the detailed histological characteristics of EC-MLD based on parameters known to be compatible with uterine MLA, including intraluminal eosinophilic secretions (Figure 2, upper panel) and various architectural patterns (7, 20). All tumors possessed intraluminal eosinophilic, hyaline-like secretions (Figure 2, lower panel). In four cases, the secretions were readily detectable throughout the tumor, whereas in the remaining cases, we only identified them occasionally. In case 4, in addition to the densely eosinophilic, inspissated secretions, we noted many different areas showing intraluminal necrotic debris, which were difficult to distinguish from hyaline-like secretions. All tumors were architecturally heterogeneous, with various combinations (Figure 3, upper panel) of tubular, ductal, papillary, retiform, glomeruloid, sex cord-like, and comedonecrosis-like patterns. These patterns frequently merged with each other. With regard to the proportion of architectural patterns, tubular and ductal patterns were the two most dominant in four cases (cases 1, 2, 3, and 5). In particular, the ductal pattern was observed in more than 50% of the tumor in three cases (cases 1, 2, and 5), whereas in one case (case 3), compactly aggregated tubules with very small lumina occupied half of the entire tumor area. The remaining case (case 4) showed that the papillary pattern was the most dominant component (45%), followed by tubular (25%) and ductal (20%). We found that all tumors harbored at least 20% of both tubular and ductal patterns. All except one case had focal (10%) solid components, leading to the diagnosis of grade 2 EC-MLD. Each of these two cases exhibited microscopic areas of sex cord-like (case 5) and comedonecrosis-like (case 3) morphologies, respectively. No retiform or glomeruloid patterns were observed. Three tumors (cases 2, 4, and 5) displayed severe nuclear pleomorphism. Conspicuous nucleoli and tumor cell necrosis were observed in two cases (cases 3 and 5). Brisk mitotic activity (more than 20 per 10 high-power fields) was noted in three cases; moreover, in case 5, atypical mitotic figures were also present. Histological features favoring EC were observed in a few cases. Scattered microscopic foci of endometrial AH/EIN (Figure 3, lower panel) were identified in two cases (cases 1 and 2). Foci of squamous differentiation (Figure 3, lower panel) were present in two cases (cases 1 and 3), whereas none of the cases showed mucinous differentiation. In case 3, several microscopic foci showing hyaline or myxoid stroma associated with clear cell features were present.

### 3.4. Immunostaining Results

**Figure 4:**
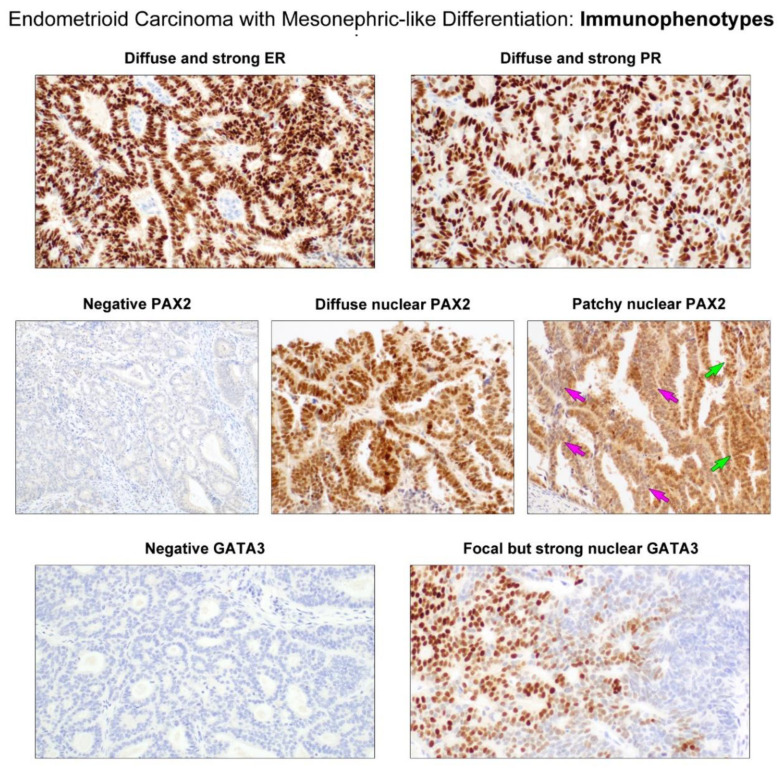
Immunostaining results. (**Upper panels**) The tumor cell nuclei display strongly positive staining for ER and PR. (**Middle panels**) PAX2 immunostaining reveals variable staining proportion: complete absence of expression (negative PAX2) and diffuse or patchy positivity. The green arrows indicate the tumor cells showing strong nuclear PAX2 expression, while those indicated by the pink arrows display weak-to-moderate cytoplasmic immunoreactivity. (**Lower panels**) Similar to PAX2 expression status, the majority of the tumor cells are negative for GATA3, but a small number of compactly aggregated tubules react strongly with GATA3.

Tumor tissue samples for immunostaining were available for all the cases. Immunostaining results are summarized in Table 4. All (5/5) EC-MLDs had moderate to strong ER expression in 50–90% tumor cells (Figure 4, upper panel). Similarly, 60–80% tumor cells expressed PR with moderate to strong staining intensity in all cases except one (4/5) (Figure 4, upper panel). In three cases, PAX2 expression was absent in >95% (2/5) or 100% (1/5) tumor cells (Figure 4, middle panel). In contrast, the remaining two cases showed moderate-to-strong nuclear PAX2 immunoreactivity in 30–70% tumor cells. In particular, 20% tumor cells in these cases exhibited intense PAX2 expression in the small tubules and glands (Figure 4, middle panel). There were abrupt transitions between the PAX2-positive and PAX2-negative areas. In some microscopic foci, individual tumor cells exhibiting strong nuclear PAX2 immunoreactivity were intermingled with those without nuclear PAX2 expression (Figure 4, middle panel). The expression pattern of GATA3 was similar to that of PAX2. All tumors were negative for GATA3 in ≥90% tumor cells (Figure 4, lower panel). Strong nuclear GATA3 immunoreactivity was observed in two cases, with staining proportions of 10% (Figure 4, lower panel) and <1%, respectively. The GATA3-positive areas consisted exclusively of compact aggregated small tubules. Additionally, the areas that were positive for PAX2 and GATA3 were negative for hormone receptors. The p53 immunostaining pattern was either the wild-type (3/5) or mutation (2/5) pattern.

Both tumors with mutant p53 expression patterns displayed diffuse and strong nuclear p53 immunoreactivity (over-expression pattern). Loss of PTEN expression was observed in three tumors. The proportion of p16 staining differed across the tumor areas. In the four p16-positive cases, nuclear and cytoplasmic p16 immunoreactivity was patchy with variable staining intensity in 20–50% tumor cells.

### 3.5. Targeted Sequencing Results

Tumor tissue samples for targeted sequencing were available for all the cases. None harbored a pathogenic v-Ki-ras2 Kirsten rat sarcoma viral oncogene homolog (*KRAS*) mutation. Two tumors (cases 2 and 5) harbored pathogenic mutations in phosphatidylinositol-4,5-bisphosphate 3-kinase catalytic subunit alpha (*PIK3CA*; c3140A > G). *PTEN* mutations were detected in two cases (missense mutation in case 2 and frameshift deletion in case 3). The truncating mutation was concordant with the loss of PTEN immunoreactivity in the latter case. Two tumors (cases 4 and 5) harbored pathogenic missense mutations in tumor protein 53 (*TP53*; c.833C > G and c.473G > T in case 4 and c.818G > T in case 5), which are concordant with p53 protein overexpression observed.

## 4. Discussion

Previous studies have indicated that the prognosis of patients with uterine MLA is significantly different from that of patients with endometrial EC [4,7,10,35]. A multi-institutional study confirmed that uterine MLA is a clinically aggressive tumor that typically presents at an advanced stage, with a predilection for recurrent pulmonary metastasis [10]. A single-institutional study also led to a similar conclusion regarding the poor outcomes of patients with MLA. Compared to EC, which is the more commonly encountered subtype of endometrial carcinoma, uterine MLA is more aggressive, with a tendency towards earlier recurrence and distant metastases [4]. Due to such differences in clinical behavior and prognosis, uterine MLA should be well-recognized and distinguished from endometrial EC upon diagnosis by pathologists. Because the majority of endometrial carcinomas are EC, pathologists should always be aware of the morphological features of uterine MLAs, so that the relatively rare MLAs are not mistaken for EC. In this background, all five cases included in this study were suspected to be MLA during the initial examination via microscopy. The following microscopic findings provided clues for this suspicion: at least 20% areas showing densely aggregated tubular structures, deep basophilia at low-power magnification because of the presence of hyperchromatic nuclei, scant cytoplasm, high nuclear-to-cytoplasmic ratio in the small tubules and glands, intraluminal eosinophilic secretions, and diverse growth patterns. All of these are classic morphological features of MLA. We considered that even though EC shows various architectural patterns, a compact proliferation of small tubules containing intraluminal eosinophilic secretions was not compatible with any known variant of EC.

Data concerning the immunostaining for ER and PR on MLA obtained in this study are quite heterogeneous, while most of the previously reported cases showed negative results [4,7,17,35]. Some authors have reported that the extent of hormone receptor positivity tends to be focal to patchy, with faint-to-weak staining intensity [5,36,37]. In this study, all five tumors showed moderate-to-strong ER immunoreactivity in ≥50% tumor cells, and four tumors demonstrated moderate-to-strong PR immunoreactivity in ≥60% tumor cells. These results support the diagnosis of EC rather than MLA. Meanwhile, positive immunoreactivity for PAX2 and GATA3 are considered characteristics of MLA. Both proteins have been used as immunohistochemical markers for determining mesonephric origin [4,7,38,39,40]. PAX2 is a protein associated with the development of the Wolffian system and is typically expressed in mesonephric tumors [14]. We recently demonstrated positive nuclear PAX2 immunoreactivity in all 11 uterine MLAs we examined [7]. In a previous study that used an immunostaining panel for MA and MLA cases [40], PAX2 was suggested as one of the markers to be included in the panel. GATA3 is also considered the best overall marker for the mesonephric lineage, given its high sensitivity and specificity [19]. This protein has been shown to be expressed in most benign and malignant mesonephric lesions, but not in the majority of endometrial and endocervical adenocarcinomas [40]. Although PAX2 and GATA3 may also stain positive in a small subset of endometrial carcinomas, including EC [38], they are mostly negative and were only focally and weakly positive in adenocarcinomas of Mullerian origin. In this study, PAX2 was negative in >95% tumor cells in three cases while GATA3 was negative in >90% tumor cells in all five cases. These results contradict the diagnosis of MLA. It is of note, however, that two tumors in this study showed strong nuclear PAX2 immunoreactivity in approximately 20% tumor cells. Furthermore, in one of these two cases, GATA3 staining was strongly positive in approximately 10% tumor cells. The areas stained positively for both PAX2 and GATA3 even consisted exclusively of a tubular architecture, closely resembling MLA. Furthermore, these areas showed a lack of ER and PR expression, complicating the differential diagnosis. These interesting findings indicate that when endometrial curettage specimens reveal some microscopic foci resembling MLA, EC-MLD could be mistaken as MLA because these areas would be at least focally positive for both PAX2 and GATA3. To avoid such misinterpretation, pathologists should also stain for ER and PR when detecting mesonephric-like areas in endometrial biopsy or curettage specimens to determine the presence of positive immunoreactivity for hormone receptors and confirm the diagnosis of EC-MLD.

In some microscopic foci, the tumor cells were positive for ER and PR but negative for GATA3 and PAX2, and vice versa. This pattern of mutually exclusive expression raised the possibility of mixed EC and MLA. However, most of the tumor cells with GATA3 and PAX2 expression demonstrated nuclear immunoreactivity for ER and PR with moderate-to-strong staining intensity. Furthermore, the tumor cells expressing both hormone receptors and mesonephric markers were intermingled with those with only one of the two. We considered single gene mutational analysis for *KRAS*, but it was almost impossible to identify and macrodissect the areas presumed to be MLA because there was no clear line of demarcation between hormonal receptor-only positive areas from mesonephric marker-only positive areas. In fact, since pathogenic *KRAS* mutations could also be detected in 20–26% of endometrial EC cases, the identification of *KRAS* mutation in MLD areas would not be helpful to confirm or exclude the possibility of mixed carcinoma.

In a single case reported by Yano et al. [41], a uterine tumor showed architectural diversity (including tubular, papillary, ductal, sieve-like, and solid patterns) and occasional small tubules and ducts lined by cuboidal-to-columnar epithelia, as well as intraluminal eosinophilic secretions. The authors designated these histological features as “mesonephric differentiation.” In this study, we used the term “MLD” for designating areas that showed classic morphological features of MLA, which are observed in endometrial EC cases. Neither a clear definition nor definitive diagnostic criteria for MLD have been described in the literature. Although it has been mentioned in several previous studies [5,6,10,11,41], no data are available on the clinicopathological and prognostic differences among endometrial EC, EC-MLD, and uterine MLA. Considering the clinical and histological characteristics of our cases, there is little evidence to suggest that EC-MLD is more aggressive than EC. Three patients were diagnosed with stage IA disease, and the other two patients were diagnosed with stage III disease. Given the information we collected, we could not determine whether the advanced stage in the latter two patients was associated with MLD. In one patient with a stage III tumor, the component that involved the adnexa did not show tubular architecture, but showed exclusively papillary growth. In the other patient, we could not determine whether the presence of MLD was attributed to the aggressive nature of their tumor, including the serosal extension and para-aortic lymph node metastasis, because it showed substantial LVSI and microcystic, elongated, and fragmented-like invasion patterns. Above all, as a limitation of this study, we could not investigate the patients’ outcomes in more depth due to the short follow-up period and small sample size. Long-term follow-up in a larger cohort is necessary to further clarify the prognostic impact of MLD in association with EC.

Several studies have documented the molecular characteristics of MLAs [7,19,24,35,42]. Most MLAs harbor pathogenic *KRAS* mutations, and some of them also have chromosomal gains in chromosomes 1 q, 10, 12, and 20. In this study, targeted sequencing analysis revealed that none of the cases harbored a pathogenic *KRAS* mutation, which is the most characteristic molecular alteration in MLA [7]. Chromosomal gain or loss was not observed; instead, all but one case harbored at least one pathogenic mutation in the *PTEN*, *PIK3CA*, or *TP53* genes. Despite their complicated histological features, the identification of mutations that are known to be commonly observed in endometrial EC, as well as the presence of wild-type *KRAS*, support the diagnosis of EC-MLD.

## 5. Conclusions

In summary, we described five cases of endometrial EC that showed the classic histological and immunohistochemical features of MLA, including a compact proliferation of small tubules and glands containing intraluminal eosinophilic secretions and focal immunoreactivity for both PAX2 and GATA3. These findings raised the possibility of uterine MLA, which is known to be more aggressive than EC. However, all tumors showed diffuse and strong immunoreactivity for hormone receptors. Furthermore, KRAS mutations, a characteristic of MLA, were not identified in any case. Instead, we detected mutations commonly observed in endometrial EC, such as in PTEN, PIK3CA, and TP53. Therefore, we diagnosed these cases as endometrial EC-MLD. Although it is important for pathologists to consider the possibility of MLA when making the diagnosis of endometrial carcinomas, ancillary tests, including immunostaining and targeted sequencing, are necessary to differentiate between EC-MLD and MLA in cases showing mesonephric-like morphology. We suggest that some morphological and immunophenotypical features, including diverse architectural patterns, compactly aggregated small tubules, glands containing intraluminal hyaline-like eosinophilic secretions, and variable degrees of PAX2 and GATA3 staining may be designated as MLD in association with common histological subtypes of endometrial carcinoma. Further investigations are necessary to elucidate the prognostic implications of this rare but distinct morphological variation in EC.

## Figures and Tables

**Table 1:** Clinical characteristics.

Case No.	Age (yr)	Previous GYN History	Presenting Symptom	MRI Finding	CA 125 (U/mL)	CA 19-9 (U/mL)	Surgery	Post-Operative Treatment	Post-Operative Local Recurrence	Post-Operative Distant Metastasis	DFS (mo)	Current Status	Alive/ Dead	OS (mo)
**1**	54	HRT	An EM mass detected on US during routine follow-up	A 2.9 cm linear EM lesion; no peritoneal seeding; no lymph node metastasis; no distant metastasis (MRI FIGO stage IA)	5.2	NA	TH + BSO + PLND	None	Absent	Absent	15	NED	Alive	18
**2**	68	None	Vaginal bleeding	A 3.3 cm EM mass with small separate lesions; no peritoneal seeding; no lymph node metastasis; no distant metastasis (MRI FIGO stage IA)	5.3	NA	TH + BSO + PLND	None	Absent	Absent	6	NED	Alive	9
**3**	57	None	Vaginal bleeding	A 5.1 cm EM mass; no peritoneal seeding; no lymph node metastasis; no distant metastasis (MRI FIGO stage IA)	29.5	NA	TH + BSO + PLND	VBT (ongoing)	NA	NA	NA	NA	NA	NA
**4**	67	Recurrent tamoxifen-related EM polyp	EM and adnexal masses detected on US during routine follow-up	A 4.5 cm EM mass; a 4.4 cm enhancing lobulating ovarian mass; no peritoneal seeding; no lymph node metastasis; no distant metastasis (MRI FIGO stage IIIA)	134.7	NA	TH + BSO + PLND + PALND	Whole pelvic RT	Absent	Absent	13	NED	Alive	16
**5**	56	None	Vaginal bleeding	A 8.8 cm EM mass with cervical extension and suspected focal serosal extension; no peritoneal seeding; no lymph node metastasis; no distant metastasis (MRI FIGO stage IIIA)	143.2	468.0	TH + BSO + PLND + PALND	CCRT (ongoing)	NA	NA	NA	NA	NA	NA

Abbreviations: GYN, gynecological; MRI, magnetic resonance imaging; HRT, hormone replacement therapy; EM, endometrium; US, ultrasonography; NA, not applicable; TH, total hysterectomy; BSO, bilateral salpingo-oophorectomy; PLND, pelvic lymph node dissection; NED, no evidence of disease; VBT, vaginal brachytherapy; PALND, para-aortic lymph node dissection; RT, radiation therapy; CCRT, concurrent chemoradiation therapy; DFS, disease-free survival; OS, overall survival.

**Table 2:** Pathological characteristics: staging parameters.

Case No	Tumor Size (cm)	Invasion Depth (cm)	Myometrial Invasion	Lymphovascular Space Invasion	Cervical Stromal Invasion	Serosal Extension	Vaginal Extension	Ovarian Extension	Salpingeal Extension	Peritoneal Metastasis	Lymph Node Metastasis	Distant Metastasis	Initial FIGO Stage
1	2.3	0.3	Less than half	−	−	−	−	−	−	−	−	−	IA
2	3.4	0.2	Less than half	−	−	−	−	−	−	−	−	−	IA
3	5.5	0.6	Less than half	+ (focal)	−	−	−	−	−	−	−	−	IA
4	4.5	0.2	Less than half	−	−	−	−	−	+ (right)	−	−	−	IIIA
5	8.5	2.3	Full thickness	+ (substantial)	−	+	−	+ (left)	−	−	+ (para-aortic)	−	IIIC2

**Table 3:** Pathological characteristics: detailed histological features.

Case No	Diagnosis	Intra-Luminal Secretion	Two Most Dominant Patterns	Proportion of Architectural Patterns						Features Favor Endometrioid Carcinoma			Nuclear Atypia			TCN
				Tub (%)	Duc (%)	Pap (%)	Solid (%)	CN-Like (%)	SC-Like (%)	AH/EIN	Squamous Metaplasia	Mucinous Metaplasia	Nuclear Pleomorphism	Conspicuous Nucleoli	Mitotic Count	
1	EC-MLD, grade 1	Frequent	Duc, Tub	25	70	5	0	0	0	+	+	+	Mild to moderate	−	2/10 HPFs	−
2	EC-MLD, grade 2	Frequent	Duc, Tub	20	70	0	10	0	0	+	−	−	Moderate to severe	−	21/10 HPFs	−
3	EC-MLD, grade 2	Frequent	Tub, Duc	50	35	0	10	0	5	−	+	−	Mild to moderate	+	14/10 HPFs	+
4	EC-MLD, grade 2	Occasional	Pap, Tub	25	20	45	10	0	0	−	−	−	Moderate to severe	−	24/10 HPFs	−
5	EC-MLD, grade 2	Frequent	Duc, Tub	35	60	0	10	5	0	−	−	−	Moderate to severe	+	25/10 HPFs	+

Abbreviations: Tub, tubular; Duc, ductal; Pap, papillary; CN-like, comedonecrosis-like; SC-like, sex cord-like; AH/EIN, atypical hyperplasia/endometrioid intraepithelial neoplasia; TCN, tumor cell necrosis; EC-MLD, endometrioid carcinoma with mesonephric-like differentiation; HPFs, high-power fields.

**Table 4:** Results of immunostaining and targeted sequencing.

Case No	Immunostaining Results																			Targeted Sequencing Results				
	ER (%)				PR (%)				PAX2 (%)				GATA3 (%)				p53	p16	PTEN Loss (%)	Gene	Mutation Type	Sequence Change	Amino Acid Change	VAF (%)
	S	M	W	N	S	M	W	N	S	M	W	N	S	M	W	N								
1	80	10	0	10	70	10	10	10	20	50	0	30	0	0	0	100	WT	Patchy	20	None				
2	50	20	5	25	50	10	15	25	0	0	<5	>95	10	0	0	90	WT	Patchy	None	*PTEN* *PIK3CA*	Missense Missense	c.389G > T c.1624G > A	p.R130L p.E542K	14.8 9.4
3	60	20	5	15	70	10	5	15	0	0	<5	>95	0	0	0	100	WT	Patchy	80	*PTEN*	Frameshift deletion	c.731delC	p.P244Lfs*12	10.4
4	40	20	0	40	<1	0	0	>99	20	10	0	70	<1	<1	0	>99	Mut (OE)	N	None	*TP53* *TP53*	Missense Missense	c.833C > G c.473G > T	p.P278R p.R158L	56.8 4.6
5	10	40	30	20	40	30	10	20	0	0	0	100	0	0	0	100	Mut (OE)	Patchy	None	*TP53* *PIK3CA*	Missense Missense	c.818G > T c.3140A > G	p.R273L p.H1047R	22.3 21.3

Abbreviations: VAF, variant allele frequency; S, strong; M, moderate; W, weak; N, negative; WT, wild-type pattern; Mut, mutation pattern; OE: overexpression.

## Data Availability

Data sharing not applicable.
